# A Faster Triphosphorylation Ribozyme

**DOI:** 10.1371/journal.pone.0142559

**Published:** 2015-11-06

**Authors:** Gregory F. Dolan, Arvin Akoopie, Ulrich F. Müller

**Affiliations:** Department of Chemistry and Biochemistry, University of California San Diego, La Jolla, California, United States of America; University of Florida, UNITED STATES

## Abstract

In support of the RNA world hypothesis, previous studies identified trimetaphosphate (Tmp) as a plausible energy source for RNA world organisms. In one of these studies, catalytic RNAs (ribozymes) that catalyze the triphosphorylation of RNA 5'-hydroxyl groups using Tmp were obtained by *in vitro* selection. One ribozyme (TPR1) was analyzed in more detail. TPR1 catalyzes the triphosphorylation reaction to a rate of 0.013 min^-1^ under selection conditions (50 mM Tmp, 100 mM MgCl_2_, 22°C). To identify a triphosphorylation ribozyme that catalyzes faster triphosphorylation, and possibly learn about its secondary structure TPR1 was subjected to a doped selection. The resulting ribozyme, TPR1e, contains seven mutations relative to TPR1, displays a previously unidentified duplex that constrains the ribozyme's structure, and reacts at a 24-fold faster rate than the parent ribozyme. Under optimal conditions (150 mM Tmp, 650 mM MgCl_2_, 40°C), the triphosphorylation rate of TRP1e reaches 6.8 min^-1^.

## Introduction

The RNA world hypothesis states that an early stage in the evolution of life used RNA as the genome and as the only genome-encoded catalyst [1–3]. This hypothesis has gained support from several directions, including the findings that RNA molecules can indeed catalyze reactions [4], that the ribosome is a catalytic RNA [5], and that deoxyribonucleotides are synthesized in cells from ribonucleotide precursors [6]. To find out how an RNA world organism could have functioned, several laboratories are trying to generate an RNA world organism in the laboratory. An important part of producing an RNA world organism is the generation of catalytic RNAs (ribozymes) that could support a self-replicating and evolving ribozyme system. Since the inception of *in vitro* selection [7, 8] and the demonstration that novel catalytic RNAs can be isolated from randomized RNA pools [9], several types of ribozymes with possible involvement in an RNA world organism have been isolated [10], most importantly a ribozyme that catalyzes RNA-dependent RNA polymerization [11]. The substrates for this ribozyme are nucleoside 5'-triphosphates; these nucleotides contain the perhaps most prebiotically plausible chemical activation group [12]. Nucleoside 5'-triphosphates can be generated from nucleosides and trimetaphosphate (Tmp) [13]. Tmp likely existed prebiotically, as suggested from the finding of large amounts of its chemical precursor in 3.5 billion year old marine sediments [14, 15]. However, the uncatalyzed triphosphorylation of nucleosides with Tmp occurs efficiently only at pH values above 12 [13], which would quickly hydrolyze the RNA polymers of an RNA world organism. We previously showed that the triphosphorylation of RNA 5'-hydroxyl groups is possible at neutral pH, with ribozymes that were obtained by *in vitro* selection [16].

**Fig 1:**
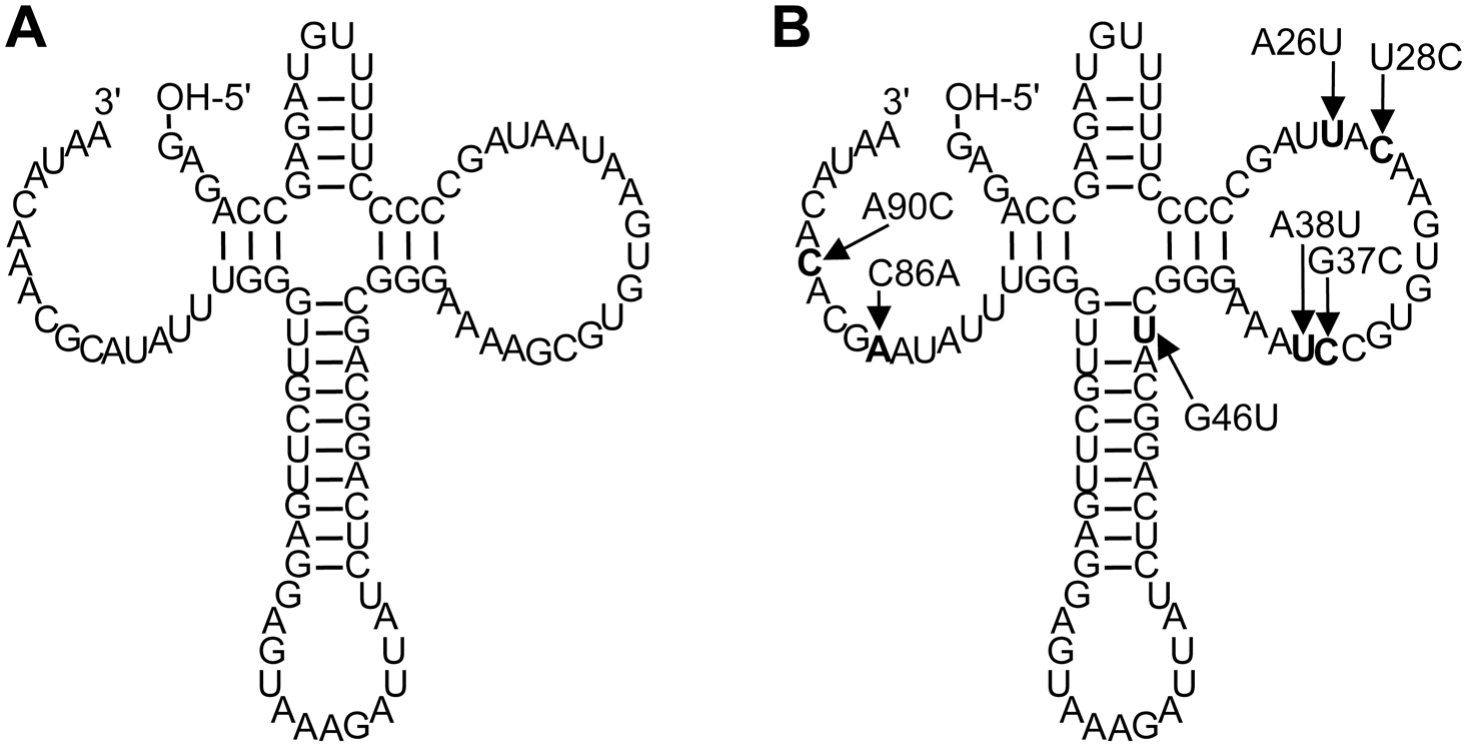
Proposed secondary structures for (A) the parent ribozyme, TPR1, and (B) the most active ribozyme identified in this study, TPR1e. TPR1e contains seven beneficial mutations relative to TPR1 (mutations are in the figure).

The only triphosphorylation ribozyme that was previously characterized in some detail, TPR1 (Fig 1A), displays single-exponential reaction kinetics with a rate of 0.013 min^-1^ at selection conditions (50 mM trimetaphosphate and 100 mM MgCl_2_ at pH 8.3) [16]. Here we searched for sequence variants of TPR1 that display faster reaction kinetics using *in vitro* selection. The most active ribozyme identified in this study, TPR1e, contains seven activity-enhancing mutations (Fig 1B) and reacts 24-fold faster than TPR1 under selection conditions. Under optimal conditions (150 mM Tmp, 650 mM MgCl_2_, 40°C), the triphosphorylation rate of TPR1e reaches 6.8 min^-1^. An additional new duplex was identified that constrains the ribozyme structure.

## Results

To identify a more active sequence variant of the previously isolated triphosphorylation ribozyme, TPR1 (Fig 1A), a 'doped selection' was performed. A partially randomized library was generated that contained the sequence of TPR1 at each position with a frequency of 76% while containing each of the other three nucleotides at a frequency of 8%. With a total of 82 nucleotides being partially randomized, around 10^3^ wild-type sequences were contained in a library of 10^13^ sequences (0.76^82^× 10^13^ ~ 1,700). The selection was performed as described previously [16] and started with an effective complexity of 7.0 × 10^13^ sequences. After two rounds of selection the number of PCR cycles required to amplify the reverse transcription product dropped from 15 to 6. Such a drop was previously found to be a good indicator that active clones dominated the library [16]. To isolate the most efficient catalysts, higher selection pressure was used in additional rounds of selection by changing the Tmp incubation conditions (shorter incubation time, lower Tmp concentration, or higher incubation temperature; S1 Fig). After three or four rounds of selection, eighty-three clones were isolated and analyzed for triphosphorylation activity (see materials and methods for details on how many clones were tested from each condition/round).

**Fig 2:**
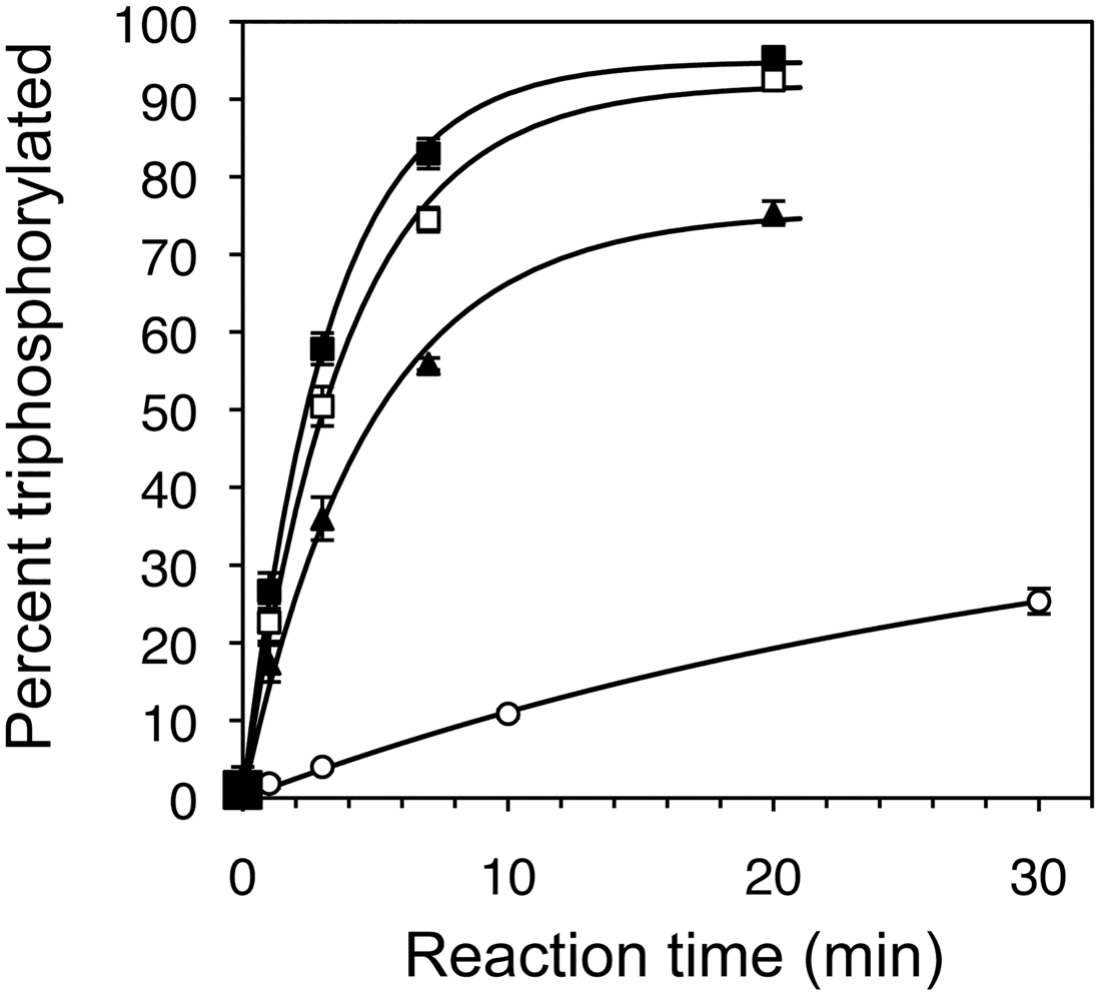
Triphosphorylation kinetics of central ribozyme variants in this study. The starting point of the doped selection was the ribozyme TPR1 (empty circles). It has a k_obs_ of 0.013 min^-1^ under selection conditions (100 mM MgCl_2_, 50 mM trimetaphosphate, 50 mM Tris/HCl pH 8.3). The most efficient isolate from the doped selection was a 16-mutation variant called clone 11 (filled triangles, k_obs_ of 0.21 min^-1^). After removal of unnecessary mutations a 5-mutation variant called TPR1-II resulted (open squares, k_obs_ of 0.25 min^-1^). Two mutations that arose independently were introduced to yield TPR1e (filled squares), a 7-mutation variant with a k_obs_ of 0.31 min^-1^. Lines are single-exponential curve fits to the data. Error bars denote the standard deviations from triplicate experiments.

Of the eighty-three sequences assayed for triphosphorylation kinetics, fifty-eight displayed triphosphorylation kinetics at least as fast as the parent ribozyme TPR1. The six fastest ribozyme clones contained the same set of two mutations (G37C, A38U), and five of them contained the mutation C86A. The fastest ribozyme was clone 11, with 16 mutations relative to TPR1 and a self-triphosphorylation rate of 0.21 ± 0.02 min^-1^ under selection conditions (50 mM Tmp, 100 mM MgCl_2_, 50 mM Tris/HCl pH 8.3) (Fig 2). This ribozyme was isolated from the selection line with decreased Tmp concentration. To identify the mutations in clone 11 that were necessary for improved triphosphorylation kinetics, all 16 mutations were individually reverted to the parent sequence (S2 Fig). Only five of the sixteen mutations were necessary for increased activity (U28C, G37C, A38U, C86A, A90C). This ribozyme was called TPR1_II. The reduction to five mutations was possible by the finding (see materials and methods) that four of the eleven nonessential mutations appeared to elongate and stabilize the long central P5 duplex of the ribozyme (U55C, A56U, U58C, U64G). These elongating mutations were only necessary in the context of two destabilizing mutations within the P5 duplex of clone 11 (A51U, C52G). All clone 11 mutations within the P5 stem-loop could be deleted while maintaining (and even increasing) catalytic activity. TPR1_II showed a self-triphosphorylation rate of 0.25 ± 0.03 min^-1^ under selection conditions (Fig 2 and S2 Fig).

To further increase triphosphorylation activity of TPR1_II the 58 clones that were at least as active as TPR1 (S3 Fig) were analyzed for statistically enriched additional mutations. Mutations were deemed enriched when they appeared with a frequency of more than 24% (a doping ratio of 24% was used for the pool design) and if they appeared preferably among the 20 most efficient ribozymes. Six such mutations were identified. Each of these mutations was individually inserted into TPR1_II. Two of the six additional mutations (A26U and G46U) showed a statistically significant increase in the rate. When both mutations were combined and inserted into TPR1_II the self-triphosphorylation rate was 0.31 ± 0.02 min^-1^ under selection conditions, 24-fold faster than TPR1 (Fig 2). Satisfyingly, the kinetics of this ribozyme also showed the highest amplitude of 95%. This suggested that the fraction of misfolded ribozyme sub-populations was reduced from ~15% (TPR1) to ~8% (TPR1_II) and finally ~5%. This variant was declared the 'winner' of the doped selection study. It contained seven mutations (A26U, U28C, G37C, A38U, G46U, C86A, A90C; Fig 1B) and was termed TPR1e.

**Fig 3:**
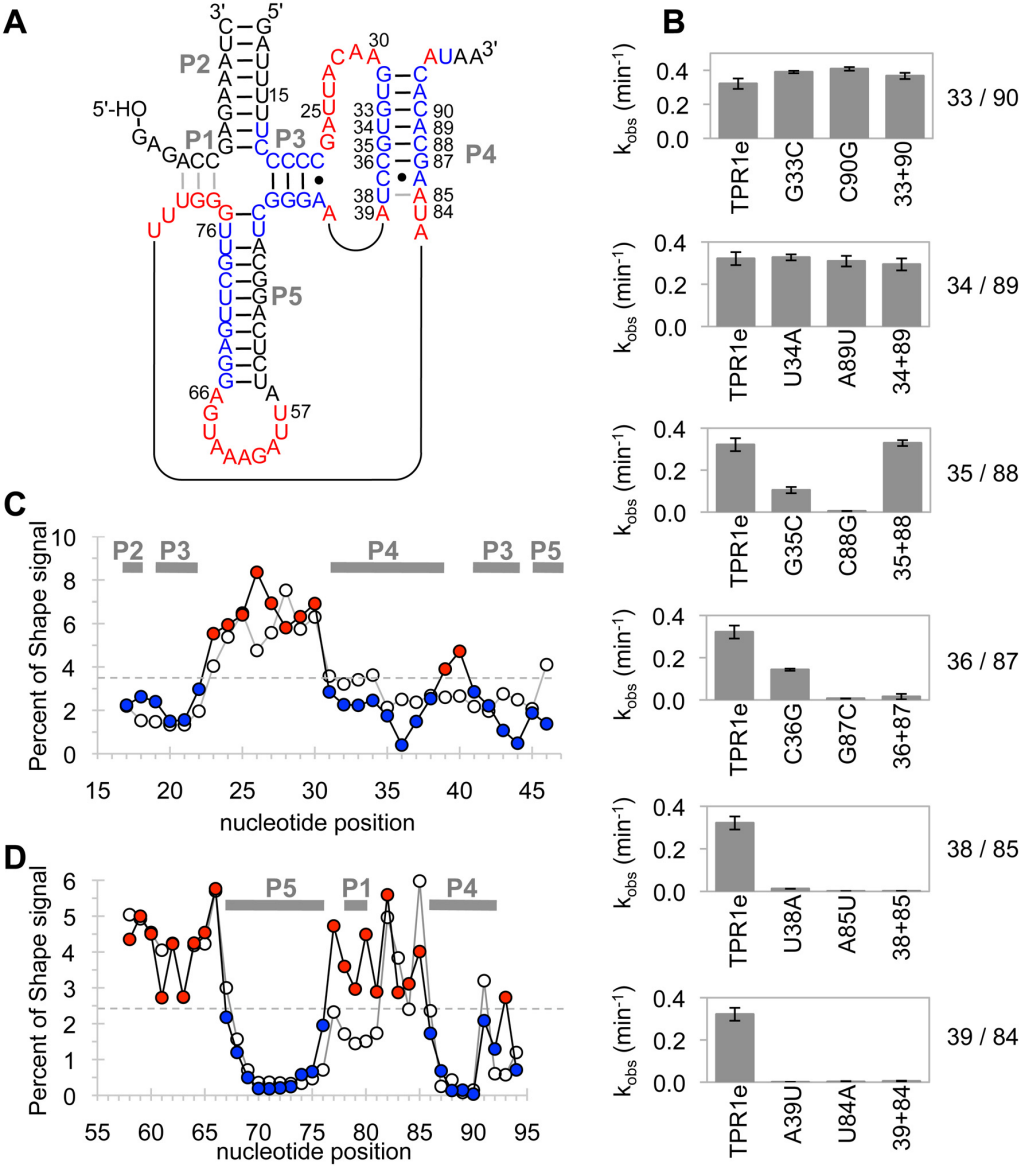
Experiments testing the formation of duplex P4. **(A)** Proposed secondary structure for ribozyme TPR1e showing proposed paired regions labeled P1-P5. All duplexes with exception of P4 were identified previously [16]. Nucleotides found accessible by Shape probing are colored in red, while protected nucleotides are shown in blue. C-A pairs are shown with a dot. The numbering scheme corresponds to the *cis*-acting ribozyme (Fig 1). Note that the duplexes P1 and P2 are formed *in trans* because the experiments were performed using the *trans* reaction. Base pairs for duplex P1 are shown in grey because for TPR1e, nucleotides 78–80 appeared accessible by Shape probing. **(B)** Apparent triphosphorylation rates of the mutated ribozymes in the base covariation experiment of twelve bases in the proposed P4 duplex. The six column graphs are in the same order as the proposed based pairs in P4 of Fig 3A. Each graph shows the triphosphorylation rate of TPR1e on the left, the two single mutants in the middle, and the double mutant on the right. Error bars are standard deviations of triplicate experiments. **(C)** Shape probing experiments for nucleotides 17 to 46 of the ribozymes TPR1 (empty circles) and TPR1e (colored circles). The normalized Shape signal is shown as a function of nucleotide position. A threshold value (grey, dashed line) is used to distinguish between low Shape reactivity (blue) and high Shape reactivity (red). The colors match the color-coding in Fig 3A. The position of duplexes P1-P5 in TPR1e is indicated by thick, solid, grey, horizontal lines. **(D)** As in (C) but for nucleotides 57 to 94 in the ribozymes.

The mutations selected in TPR1e led to the complementarity between eight bases in nucleotides 31–39 and eight bases in nucleotides 84–92, interrupted only by a single C:A pair (Fig 3A). This suggested the existence of a previously unrecognized duplex, P4. To test whether this duplex formed, base covariation experiments were conducted at six positions in the expected duplex, testing for base pairs G33/C90, U34/A89, G35/C88, C36/G87, U38/A85, and A39/U84 (Fig 3B). The drop in activity of individual mutations G35C and C88G was rescued by the double-mutation suggesting that this portion of the P4 duplex is formed in the active ribozyme. No changes in activity were observed with the mutations of G33C and C90G and with mutations U34A and A89U, suggesting that these bases were not involved in the duplex or that their contribution to duplex stability was not necessary for full activity of the ribozyme. Double mutations at the positions 36/87, 38/85, and 39/84 did not rescue the inhibitory effects of the single mutations, suggesting that the identity of these bases was important for activity. In summary, the base covariation experiments of the proposed P4 duplex were inconclusive because only one out of six positions showed the characteristic deleterious effects of single mutations coupled to a rescue effect of the double mutation.

To interrogate the proposed duplex with a different method, SHAPE probing experiments with 1-methyl-7-nitroisatoic acid (1M7) were conducted [17]. This compound specifically reacts with 2'-hydroxyl groups of flexible nucleotides such that positions of low reactivity correlate with the presence of double strands. Both TPR1 and TPR1e were subjected to the same analysis. The accessibility data between nucleotides U17 and U46 were consistent with the position of the P2, P3, and P5 helix. Additionally the protected region from G31 to U38 suggested the existence of the P4 duplex (Fig 3C). While positions U38/A85 and A39/U84 would be complementary to stack upon the P4 duplex these base pairs do not appear to form in TPR1 or TPR1e (Fig 3C). Importantly, the protection of nucleotides A86 to C92 confirmed the existence of the duplex (Fig 3D). The P1 duplex that appears to exist in TPR1 (Fig 3D, position 78–80, [16]) does not appear to exist in TPR1e. Interestingly, two of the most important activity-enhancing mutations identified by the doped selection (G37C and C86A) led to a C:A pair at the end of the proposed P4 duplex. In summary, the chemical probing experiments by 1M7 confirm the existence of the P4 duplex, are consistent with previous 1M7 probing of TPR1, and suggest distinct structural changes between TPR1 and TPR1e. The newly identified P4 duplex generates strong structural constraints and suggests a very compact three-dimensional structure for the ribozyme.

The parent ribozyme TPR1 did not show detectable triphosphorylation of free nucleosides [16]. To test whether TPR1e was able to triphosphorylate free nucleosides, ^14^C labeled guanosine was incubated with eight different variants of TPR1e. Unfortunately, no triphosphorylation of free nucleoside was detected with any of eight variants of TPR1e tested (data not shown). Eight ribozyme variants were tested because the ribozyme 5'-terminus could have hindered binding of the free nucleoside. In addition to the full-length TPR1e, five variants had their 5'-terminus truncated by 1, 2, 3, 6, or 18 nucleotides; all of these ribozymes contained 5'-hydroxyl groups. Additionally, the ribozyme truncated by 1 nucleotide was also tested with a 5'-phosphate and a 5'-triphosphate. So far no ribozyme has been identified that can triphosphorylate free nucleosides.

**Fig 4:**
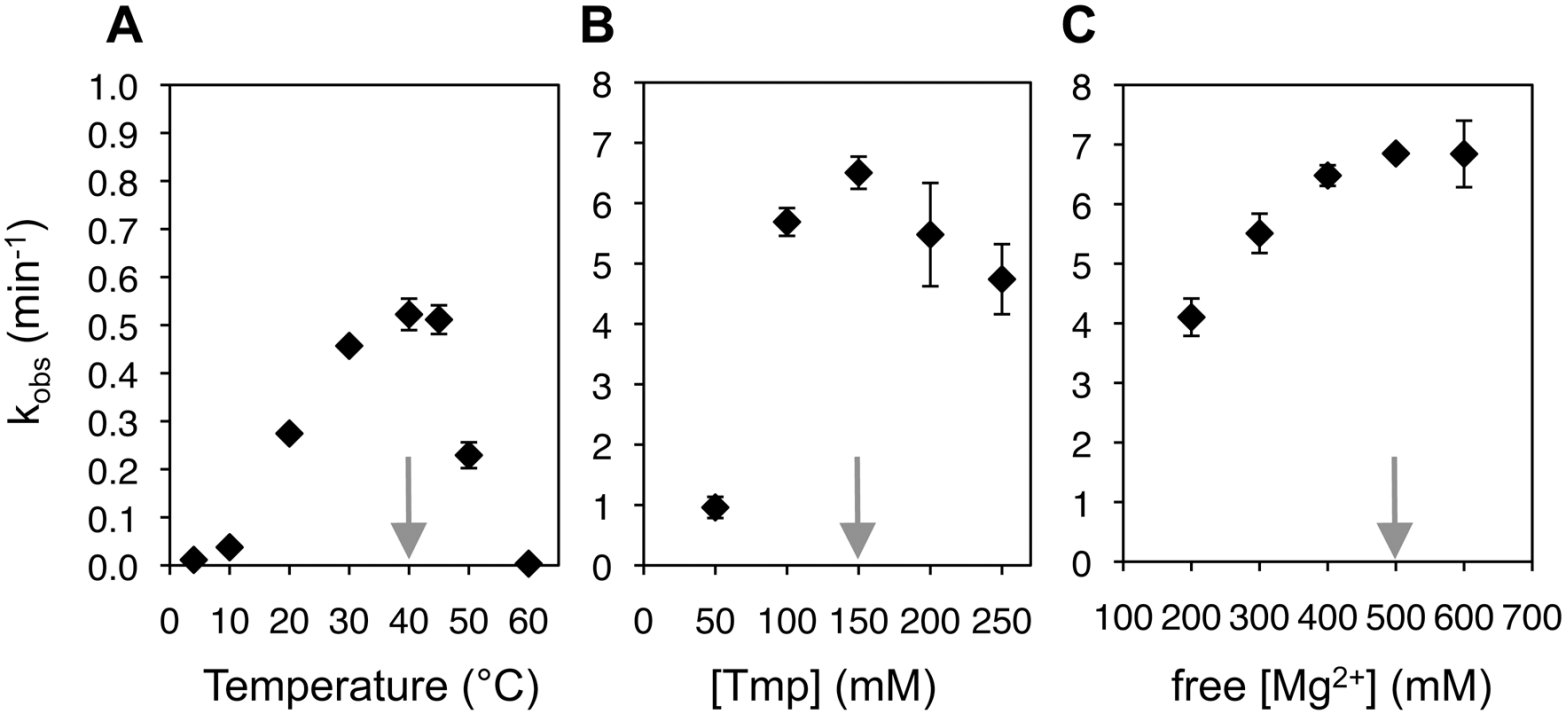
Determination of optimal TPR1e triphosphorylation conditions. **(A)** Observed triphosphorylation rate as function of the temperature, at 50 mM Tmp, 100 mM MgCl_2_, and 50 mM Tris/HCl pH 8.3 **(B)** Influence of the trimetaphosphate concentration on the observed reaction kinetics at 40°C, and with an excess of 400 mM MgCl_2_ over Tmp. **(C)** Influence of the free Mg^2+^ concentration on the triphosphorylation rate at 40°C and 150 mM Tmp. The free Mg^2+^ concentration is the total Mg^2+^ concentration minus the concentration of Tmp because each Tmp appears to be coordinated by one Mg^2+^ at these concentrations and pH 8.3 [16]. The grey arrows indicate the optimum condition for each series of experiments. Note that the scale in (A) is different from the scale in (B) and (C). Error bars are standard deviations of triplicate experiments.

**Fig 5:**
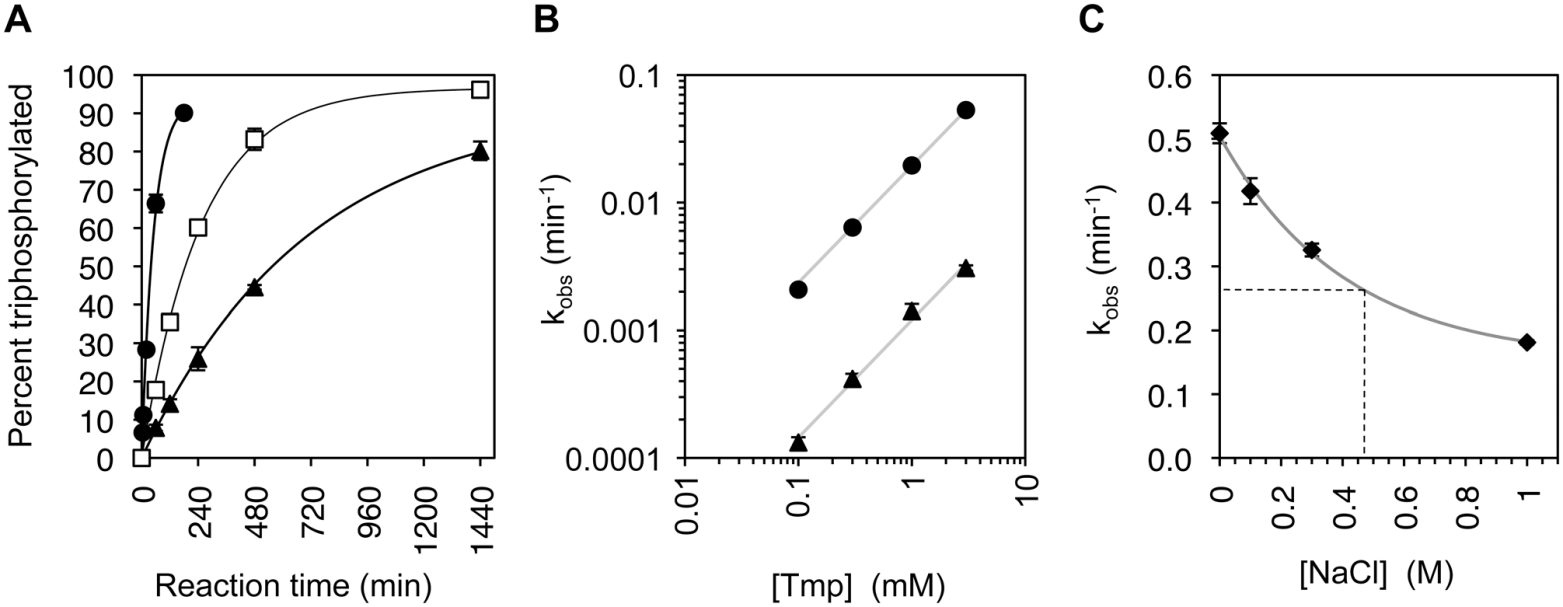
Triphosphorylation kinetics of TPR1e. **(A)** At a Tmp concentration of 1 mM, the triphosphorylation kinetics are shown for synthetic seawater at 22°C (black triangles, k_obs_ = 0.0014 min^-1^, max = 93%), and at 40°C (empty squares, k_obs_ = 0.0039 min^-1^, max = 97%). For comparison, the reaction kinetics are shown for 54 mM MgCl_2_ in Tris/HCl pH 8.3 at 22°C (black circles, k_obs_ = 0.020 min^-1^, max = 93%). This latter condition lacks all seawater components with exception of Mg^2+^. Error bars are standard deviations from triplicate experiments, and are smaller than the symbols if not visible. Curves are single-exponential fits to the data. **(B)** Titration of the Tmp concentration in the reaction at 22°C, in synthetic seawater (black triangles) and in 54 mM MgCl_2_ with 50 mM Tris/HCl pH 8.3 (black circles). The offset between the linear fits (grey lines) is 15-fold, on average. **(C)** Titration of sodium chloride into a triphosphorylation ribozyme reaction containing 50 mM Tmp and 140 mM MgCl_2_. The grey line is a single-exponential fit to the data (with offset) and identifies a 1.9-fold reduction in k_obs_ at 470 mM [NaCl], the same NaCl concentration as in synthetic seawater (dashed line). Error bars are standard deviations from triplicate experiments, and are smaller than the symbols if not visible.

To identify the optimal reaction conditions for the evolved ribozyme (TPR1e), the influence of temperature, Tmp concentration, and Mg^2+^ concentration were analyzed sequentially. First, the temperature was varied between 5°C and 60°C, and an optimum at 40°C was determined (Fig 4A). At 40°C, the optimal Tmp concentration was at 150 mM (Fig 4B), and the optimal concentration of free Mg^2+^ at 150mM Tmp was at 500 mM (Fig 4C). Interestingly, the dependence on the Tmp concentration at these high Mg^2+^ conditions and high temperature showed a cooperative effect, with a significantly higher rate at 100 mM Tmp (5.7 min^-1^) than what would result from doubling the rate at 50 mM Tmp (2 • 0.96 min^-1^ = 1.92 min^-1^). This suggested that a second molecule of trimetaphosphate binds to the ribozyme and/or the first trimetaphosphate molecule and increases the reaction rate. This effect was not observed with the parent ribozyme TPR1 [16]. The mechanism of the cooperative effect is currently unclear. Notably, the effect did not appear at more modest Mg^2+^ concentrations (~50 mM free Mg^2+^ versus 400 mM free Mg^2+^) and lower temperature (22°C vs. 40°C), where a linear correlation between Tmp concentration and reaction rate was observed (Fig 5 and [16]). At the optimal conditions (40°C, 150 mM Tmp, 650 mM total MgCl_2_, 50 mM Tris/HCl pH 8.3) the cooperative effect afforded a reaction rate of 6.8 min^-1^.

To test whether TPR1e showed RNA triphosphorylation activity under prebiotically more plausible conditions, the triphosphorylation reaction was performed in synthetic seawater with 1 mM Tmp (Fig 5A). The concentration of 1 mM Tmp was considered to be in the upper range of prebiotically plausible concentrations [15]. The synthetic seawater contained 470 mM Na^+^, 550 mM Cl^-^, 28 mM SO_4_^2-^, 54 mM Mg^2+^, 10.5 mM Ca^2+^, 10.1 mM K^+^, 2 mM HCO_3_^-^, and 0.3 mM CO_3_^2-^ [18, 19]. The triphosphorylation rates under these conditions were 0.0014 min^-1^ at 22°C and 0.004 min^-1^ at 40°C. The rate in synthetic seawater, which contains 54 mM MgCl_2_, was compared to the rate with 54 mM MgCl_2_ in the absence of the other components of synthetic seawater. At Tmp concentrations between 0.1 mM to 3 mM, the rate was consistently about 15-fold lower in synthetic seawater (Fig 5B). The contribution of sodium chloride to this inhibition (470 mM in synthetic seawater) was about 2-fold (dashed line in Fig 5C). This showed that TPR1e is inhibited by several components of synthetic seawater, and that the evolved ribozyme TPR1e is able to triphosphorylate with a rate of 0.004 min^-1^ under prebiotically plausible conditions.

## Discussion

In the present study, a ribozyme catalyzing the triphosphorylation of RNA 5'-hydroxyl groups with Tmp was subjected to a doped selection, which resulted in the improved ribozyme, TPR1e. A previously unidentified duplex was identified in this ribozyme, indicating a very compact structure. TPR1e shows triphosphorylation kinetics of 6.8 min^-1^ under optimal conditions. In synthetic seawater with 1 mM Tmp at 40°C, a triphosphorylation rate of 0.004 min^-1^ was measured.

The concentration of 1 mM Tmp appears to be in a prebiotically plausible range because the prebiotic steady-state concentration of polyphosphates was estimated to have been 10 nM—10 μM in the mixed zone of the ocean, with localized concentrations across the surface of the Earth in excess of 1 mM [15]. The detection of TPR1-catalyzed triphosphorylation in synthetic seawater with 1 mM Tmp is encouraging; however, the specific ribozyme presented here would most likely not have been useful to RNA world organisms for two reasons. First, TPR1e did not show detectable triphosphorylation of free nucleosides; an activity that would have generated nucleoside 5'-triphosphates that can be used in RNA polymerization [11]. Second, while TPR1e can act in trans on short RNA oligonucleotides (Fig 3), it did not mediate multiple turnover reactions (data not shown). Multiple-turnover triphosphorylations of short RNA oligonucleotides could have been sufficient to mediate RNA polymerization if RNA polymerization would have proceeded in 3'-5' direction instead of today's biological 5'-3' direction [10, 11]. Future developments may generate ribozymes that catalyze multiple turnover triphosphorylation of RNA oligonucleotides and / or the triphosphorylation of free nucleosides.

The results of the doped selection can be helpful for the design of other doped selection experiments. The doping ratio used in this work (24% randomization of the wild type sequence) together with the complexity of the pool (7 × 10^13^) showed that the sequence space of single, double, and triple mutants of the parent ribozyme (TPR1) was sampled completely, and quadruple mutants were sampled to ~77% (P = 1-(1–0.76^78^× 0.08^4^)^(7×10^13^) = 0.77). This doping ratio was chosen in an attempt to maximize sequence coverage without losing the ribozyme pool’s catalytic activity, but two observations from this work suggest that a lower doping rate would likely have been more beneficial. First, the most active ribozyme from the selection contained several mutations that destabilized stem P5 and required additional mutations to overcome the destabilizing effects. Second, the number of mutations per ribozyme was an average of 19.8 in the starting pool (based on 10 sequences) while the selected ribozymes displayed an average of 12.2 mutations per ribozyme (based on 82 sequences; S4 Fig). Collectively, the presence of inhibiting mutations in even the most active clones and the preferential selection of ribozymes with fewer total mutations indicates that a lower doping ratio would likely have been more beneficial.

## Materials and Methods

### Design of the doped pool

The doped pool was generated from a 113 nucleotide long DNA oligonucleotide (Sigma-Aldrich) based on the original TPR1 sequence [16]. The composition of bases at each position of TPR1 was the original sequence to 76% and the other three nucleotides to 8% each. The first 14 nucleotides of TPR1 were kept constant and an additional 12 nucleotides were added to the 3' end to be used as 5' and 3' primer binding sites during the selection. Hand-mixed phosphoramidites were used by Sigma at each partially randomized position to achieve the desired 76% to 8% ratio (confirmed by sequencing of 10 clones; data not shown). The hammerhead ribozyme and T7 promoter necessary for performing the selection procedure had the same sequence as previously [16], and were added as 5'-primer during PCR.

### Selection

The initial pool for the selection contained an effective population size of 7×10^13^ unique sequences. The protocol of the selection was identical to our previous selection [16]. During the first round of the selection the incubation with Tmp lasted 3 hours, in the presence of 50 mM Tmp, 100 mM MgCl_2_, and 50 mM Tris/HCl pH 8.3, at a temperature of 22°C. Subsequent rounds of the selection were split into three lines with different selection pressures (S1 Fig). In line one the incubation time with Tmp was decreased from 3 hours, to 2 minutes, then 20 seconds. In line two the concentration of Tmp was decreased from 50 mM, to 5 mM, then 1 mM. In line three the incubation temperature was increased from 22°C, to 42°C, then 50°C. A total of eighty-three ribozymes were then tested for activity, with 53 clones from line 1 round 3, ten clones from line 1 round 4, ten clones from line 2 round 3, and ten clones from line 3 round 3. The six fastest ribozyme clones were identified from all lines but line 3 (increased temperature). The fastest clone, clone 11, came from line 2 round 3 (decreased Tmp concentration).

### Kinetic analysis of *cis*-triphosphorylation reactions

The ribozyme clones were prepared as described previously [16]. PCR products from initial isolates of the selection were cloned into pUC19, downstream of a hammerhead ribozyme and T7 promoter. After PCR amplification of this ribozyme cassette, ribozymes were obtained by run-off transcription from PCR products using T7 RNA polymerase. Ribozymes were internally labeled using α[^32^P]-ATP during T7 transcription. The hammerhead ribozyme cleaves co-transcriptionally and generates a 5'-hydroxyl group on the triphosphorylation ribozymes. The ribozymes were separated by denaturing polyacrylamide gel electrophoresis (PAGE), excised by UV shadowing, eluted in 300 mM NaCl, ethanol precipitated, dissolved in 10mM Tris/HCl pH 8.3 and their concentration measured by the A_260_. All isolates were initially tested with the additional twelve nucleotides used as a 3'-PCR primer-binding site during the selection. Subsequent experiments with clone 11 to identify the functional mutations also employed this protocol with the exception that the twelve 3'-terminal nucleotides were removed.

The activity assay was performed as described previously [16]. In short, 8 μM ribozyme was incubated at 22°C with 50 mM Tmp, 100 mM MgCl_2_, and 50 mM Tris/HCl pH 8.3. Small aliquots were taken at different time points and quenched with an excess of Na_2_EDTA. DNAzyme reactions to separate the eight 5'-terminal nucleotides of the ribozyme were initiated by addition of DNAzyme and a 24-nucleotide long DNA oligonucleotide, both at a final concentration of 1.6μM and two-fold above the ribozyme concentration. The 24-nucleotide long DNA oligonucleotide annealed immediately downstream of the DNAzyme to the triphosphorylation ribozyme and assisted in DNAzyme binding and cleavage as well as inactivating the triphosphorylation ribozyme. Note that for the faster ribozymes of this study the quenching required perfect complementarity between the 24-nucleotide DNA and the corresponding sequence of the ribozyme. After heat renaturation (2'/80°C), MgCl_2_ was added to a final concentration of 100 mM to allow DNAzyme catalysis (1h/37°C). Aliquots of the DNAzyme reaction were quenched in formamide and Na_2_EDTA. Samples were run on a 22.5% denaturing PAGE gel to separate the triphosphorylated and unreacted 8-nucleotide fragments resulting from the DNAzyme reaction. Bands were imaged on a PMI phosphoimager (BioRad) and quantified using the Quantity One software. Triplicate experiments used one, two, or three different preparations of the ribozyme. The variability between different ribozyme preparations was smaller than the day-to-day variability of the experiments and therefore did not significantly affect the errors.

### Kinetic analysis of *trans*-triphosphorylation reactions

All experiments with TPR1e used the *trans*-triphosphorylation protocol and performed essentially as shown [16]. In the *trans*-triphosphorylation experiment, an internally radiolabeled 14-nucleotide RNA oligomer with a 5' hydroxyl group was used as substrate. The 5' portion of the ribozyme was truncated to anneal to the 14-nucleotide substrate via the P1 and P2 helices and position the substrate 5'-hydroxyl group at the active site. Ribozymes were transcribed using T7 RNA polymerase without radioactive label and purified as described above. The substrate oligonucleotide was prepared essentially as previously [16]. Briefly, a PCR product was generated that encoded a T7 promoter, and the substrate flanked by hammerhead ribozymes at their 5'- and 3'-terminus. The hammerhead ribozymes were used to generate the 5'-hydroxyl group and to ensure homogeneity at the 3'-terminus of the substrate. T7 transcription was performed with α-^32^P-ATP. T7 transcripts were purified as described for the ribozymes above. Trace amounts of substrate were used in each reaction.

The triphosphorylation experiment was set up similar to the *cis*- reaction, where ribozyme and substrate were mixed with Tmp, MgCl_2_, and Tris/HCl pH 8.3 (or other components as given in the text). Samples were taken by adding a small aliquot from the reaction into formamide and EDTA. The *trans*-reaction was quenched efficiently with the addition of formamide. Reaction products were run on a 20% PAGE gel to separate the triphosphorylated from unreacted 14-nucleotide substrate. The *trans*-reaction was used in the covariation, simulated seawater, NaCl titration, and optimal conditions experiments.

### Mutagenesis for the identification of beneficial mutations, and base covariation experiments

Two strategies were used for generating the different ribozyme constructs, PCR mutagenesis and quickchange site-directed mutagenesis [20]. Mutations reverting the sequence of clone 11 to the wild type sequence were introduced by site-directed mutagenesis for the 12 internal mutations while the four mutations near the ribozyme 3'-terminus were introduced with a 3'-PCR primer. Internal mutations in TPR1_II and its variants were introduced by overlapping PCR primers that contained the necessary mutations. PCR products were then cloned into pUC19 and sequenced. The six mutations inserted into TPR1_II were introduced using quickchange site directed mutagenesis. For the covariation experiment, positions G33C and G35C were introduced using quickchange site directed mutagenesis; positions C90G and C88G were introduced via a 3'-PCR primer.

To reduce the number of mutations in TPR1 clone 11 to a minimum, we hypothesized that two mutations (A51U, C52G) destabilized the P5 stem, and that this effect was compensated by four additional mutations in the P5 loop (U55C, A56U, U58C, U64G) that appeared to extend the P5 stem. Indeed, when both types of mutations were removed from clone 11 (in addition to A63C and U76C), the activity of the resulting ribozyme (TPR1_II) matched and even exceeded that of clone 11.

### SHAPE probing experiments

The Shape probing experiments were conducted essentially as described [16, 17]. Briefly, 1 μM trans-acting ribozyme was incubated with 1.5 μM of its 14-nucleotide substrate and 2 mM 1M7 [21] in a solution of 100 mM MgCl_2_, 50 mM trisodium Tmp and 50 mM HEPES/NaOH pH 8.0 for 3 minutes. 1M7 was added as a 20 mM solution in DMSO, leading to a final DMSO concentration of 10% (v/v). Products were ethanol precipitated and reverse transcribed with 5'-[^32^P] radiolabeled primers and Superscript III reverse transcriptase (Invitrogen). The region from U17 to U46 was analyzed with a reverse transcription primer complementary to nucleotides G68—C48. For analysis of the region U57 and U94 the ribozymes were extended at their 3'-terminus such that a 12-nucleotide reverse transcription primer could facilitate analysis of the sequence close to the ribozyme 3'-terminus. Products were separated by denaturing 15% PAGE, exposed to phosphorimager screens, scanned, and quantitated with the software Quantity One. Subtraction of the band intensities from a reaction containing DMSO without 1M7 from the intensities of the reaction containing 20 mM 1M7 in DMSO gave the SHAPE reactivities at each position. Because the reverse transcription efficiency of TPR1 was reproducibly lower than that of TPR1e the SHAPE signals were normalized to the total radioactivity in the analyzed region as shown in Fig 3C and 3D.

## Supporting Information

S1 Fig Diagram of doped selection performed. Boxes represent pools of RNA and horizontal arrows represent rounds of selection. From the starting pool of the doped selection (R0 pool, left), round 1 was performed using ‘standard’ reaction conditions. 'Standard' describes the incubation conditions that were used in the previous selection, with an incubation time of 3 hours, an incubation temperature of 22°C, a ribozyme concentration of 100 nM, a Tmp concentration of 50 mM, a MgCl_2_ concentration of 100 mM, and 50 mM Tris/HCl pH 8.3. For selection steps under different conditions, only those conditions that differed from the standard condition are noted below the arrow. Round 2 was performed using three separate conditions (lines), as indicated by the branching of the R1 pool. Line 1 maintained ‘standard’ conditions in round 2, while Line 2 lowered the Tmp concentration while maintaining the same free Mg^2+^ concentration (5mM TmP / 55mM MgCl_2_, as indicated under the arrow), and Line 3 used ‘standard’ conditions at an elevated temperature (42^°^C, as indicated under the arrow). In subsequent rounds each line was kept separate & exposed to the conditions indicated below the arrows. Blue labels indicate pools where individual ribozymes were cloned, isolated, and tested for activity.
(TIF)

S2 Fig Identification of mutations in isolated ribozyme clone 11 that are necessary and sufficient for full activity. Mutated nuclotides are labeled and shown in bold. **(A)** Proposed secondary structure for clone 11, which contains 16 mutations relative to the parent ribozyme TPR1 (labeled with arrows). **(B)** Observed single-exponential rates of triphosphorylation kinetics with ribozyme variants of clone 11 in which the indicated mutation is reverted to the parent sequence. The shown results are from single measurements; experimental errors were in the range of 0.02 min^-1^. **(C)** Observed amplitudes of the single-exponential reaction kinetics of the same clones. The shown results are from single measurements; experimental errors were in the range of 3%. **(D)** Proposed secondary structure of ribozyme variant TPR1_II, which contains the five necessary mutations of clone 11.
(TIF)

S3 Fig Position of mutations in the 58 clones isolated from the selection that had at least the same activity as the parent ribozyme TPR1. The number on the x-axis denotes the nucleotide position in the ribozyme. For position 1–14 (shaded box) no mutation data are available because this was the primer binding site during the selection. The columns are coded according to the nucleotide to which the position was mutated: dark grey: mutation to C; white: mutation to U; black: mutation to G; light grey: mutation to A. White triangles denote the 16 mutations present in clone 11. Grey triangles denote additional mutations that were tested for benefits by inserting them into ribozyme variant TPR1_II. Black triangles indicate the seven mutations of TPR1e. The illustration below the graph shows the secondary structure elements in the ribozyme.
(TIF)

S4 Fig Mutational load in the pool before and after selection. The clones identified in the doped selection were binned according to their number of mutations (including deletions). The number of mutations is given on the x-axis; the number of clones per bin is given on the y-axis. Clones isolated from the pool before selection are represented in black columns. Clones isolated after selection are shown in grey columns. A total of 10 clones were analyzed from the pre-selected pool; a total of 82 clones was analyzed after the selection. The average number of mutations in clones before the selection is 19.8; after the selection it is 12.2.
(TIF)
