# Supplementary figures and images for: A Faster Triphosphorylation Ribozyme
Source: PLoS One. 2015 Nov 6;10(11):e0142559. doi: 10.1371/journal.pone.0142559 (PMC4636267; doi:10.1371/journal.pone.0142559)

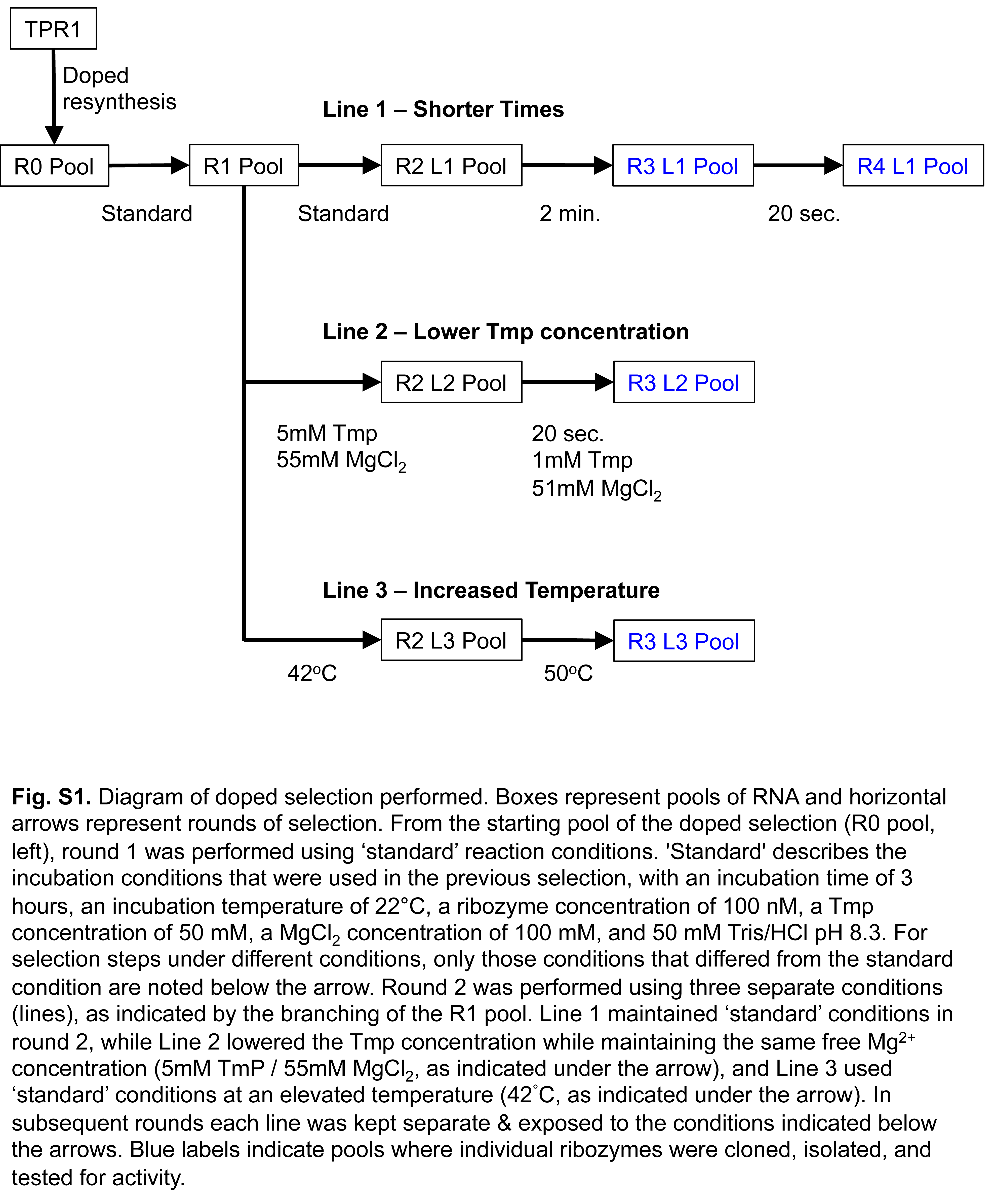

Supplement: S1 Fig — Boxes represent pools of RNA and horizontal arrows represent rounds of selection. From the starting pool of the doped selection (R0 pool, left), round 1 was performed using ‘standard’ reaction conditions. 'Standard' describes the incubation conditions that were used in the previous selection, with an incubation time of 3 hours, an incubation temperature of 22°C, a ribozyme concentration of 100 nM, a Tmp concentration of 50 mM, a MgCl2 concentration of 100 mM, and 50 mM Tris/HCl pH 8.3. For selection steps under different conditions, only those conditions that differed from the standard condition are noted below the arrow. Round 2 was performed using three separate conditions (lines), as indicated by the branching of the R1 pool. Line 1 maintained ‘standard’ conditions in round 2, while Line 2 lowered the Tmp concentration while maintaining the same free Mg2+ concentration (5mM TmP / 55mM MgCl2, as indicated under the arrow), and Line 3 used ‘standard’ conditions at an elevated temperature (42°C, as indicated under the arrow). In subsequent rounds each line was kept separate & exposed to the conditions indicated below the arrows. Blue labels indicate pools where individual ribozymes were cloned, isolated, and tested for activity. (TIF) [file pone.0142559.s001.tif]

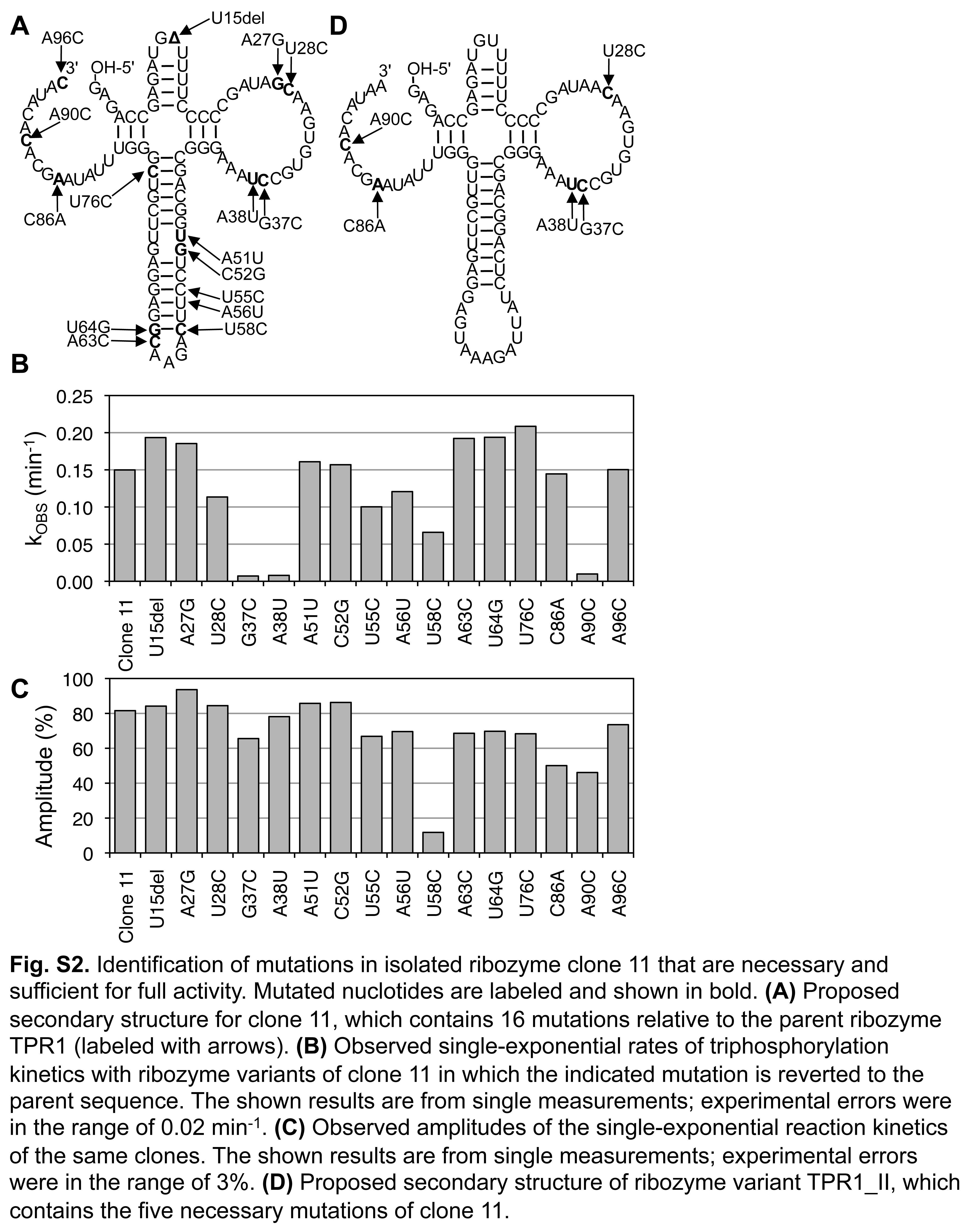

Supplement: S2 Fig — Mutated nuclotides are labeled and shown in bold. (A) Proposed secondary structure for clone 11, which contains 16 mutations relative to the parent ribozyme TPR1 (labeled with arrows). (B) Observed single-exponential rates of triphosphorylation kinetics with ribozyme variants of clone 11 in which the indicated mutation is reverted to the parent sequence. The shown results are from single measurements; experimental errors were in the range of 0.02 min-1. (C) Observed amplitudes of the single-exponential reaction kinetics of the same clones. The shown results are from single measurements; experimental errors were in the range of 3%. (D) Proposed secondary structure of ribozyme variant TPR1_II, which contains the five necessary mutations of clone 11. (TIF) [file pone.0142559.s002.tif]

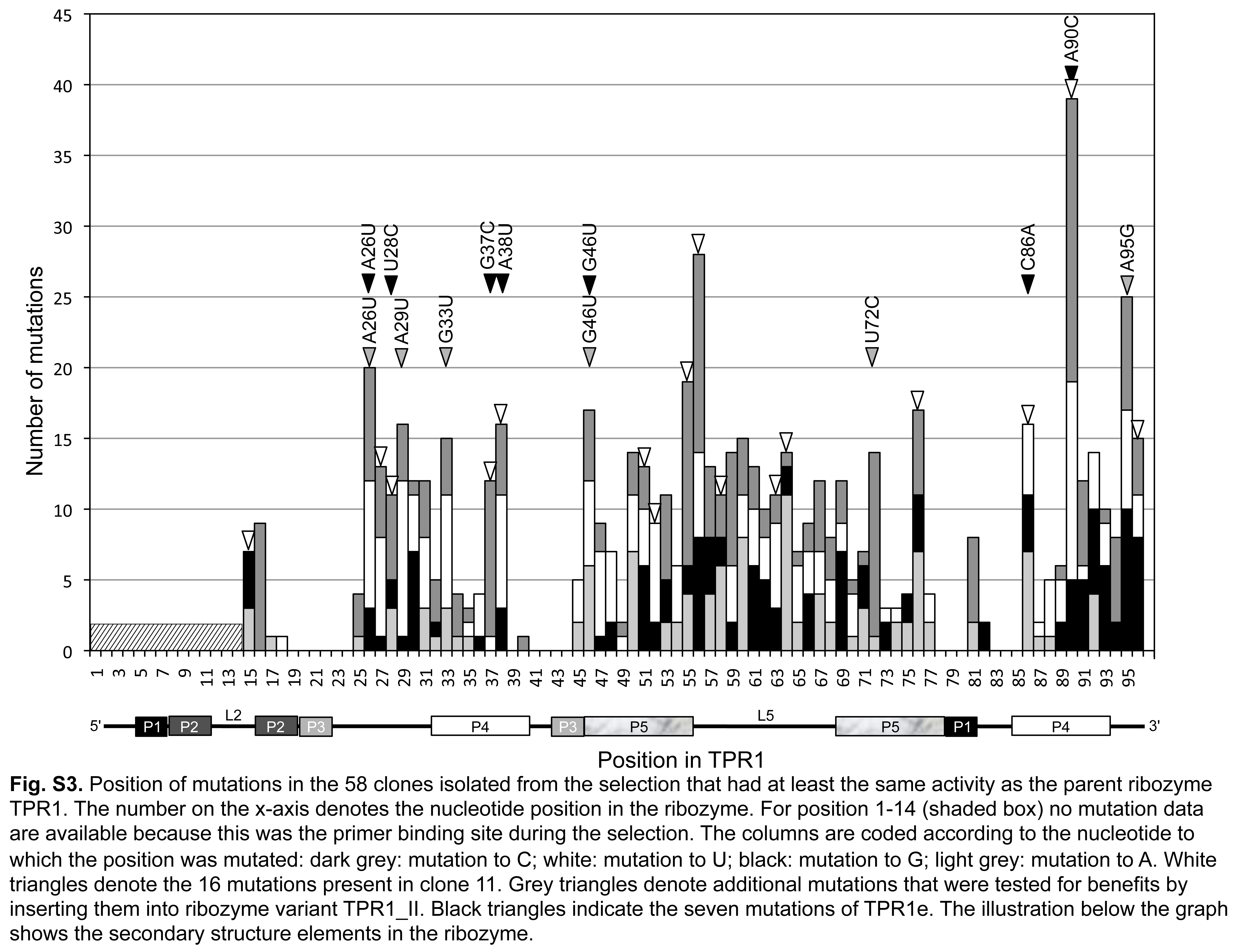

Supplement: S3 Fig — The number on the x-axis denotes the nucleotide position in the ribozyme. For position 1–14 (shaded box) no mutation data are available because this was the primer binding site during the selection. The columns are coded according to the nucleotide to which the position was mutated: dark grey: mutation to C; white: mutation to U; black: mutation to G; light grey: mutation to A. White triangles denote the 16 mutations present in clone 11. Grey triangles denote additional mutations that were tested for benefits by inserting them into ribozyme variant TPR1_II. Black triangles indicate the seven mutations of TPR1e. The illustration below the graph shows the secondary structure elements in the ribozyme. (TIF) [file pone.0142559.s003.tif]

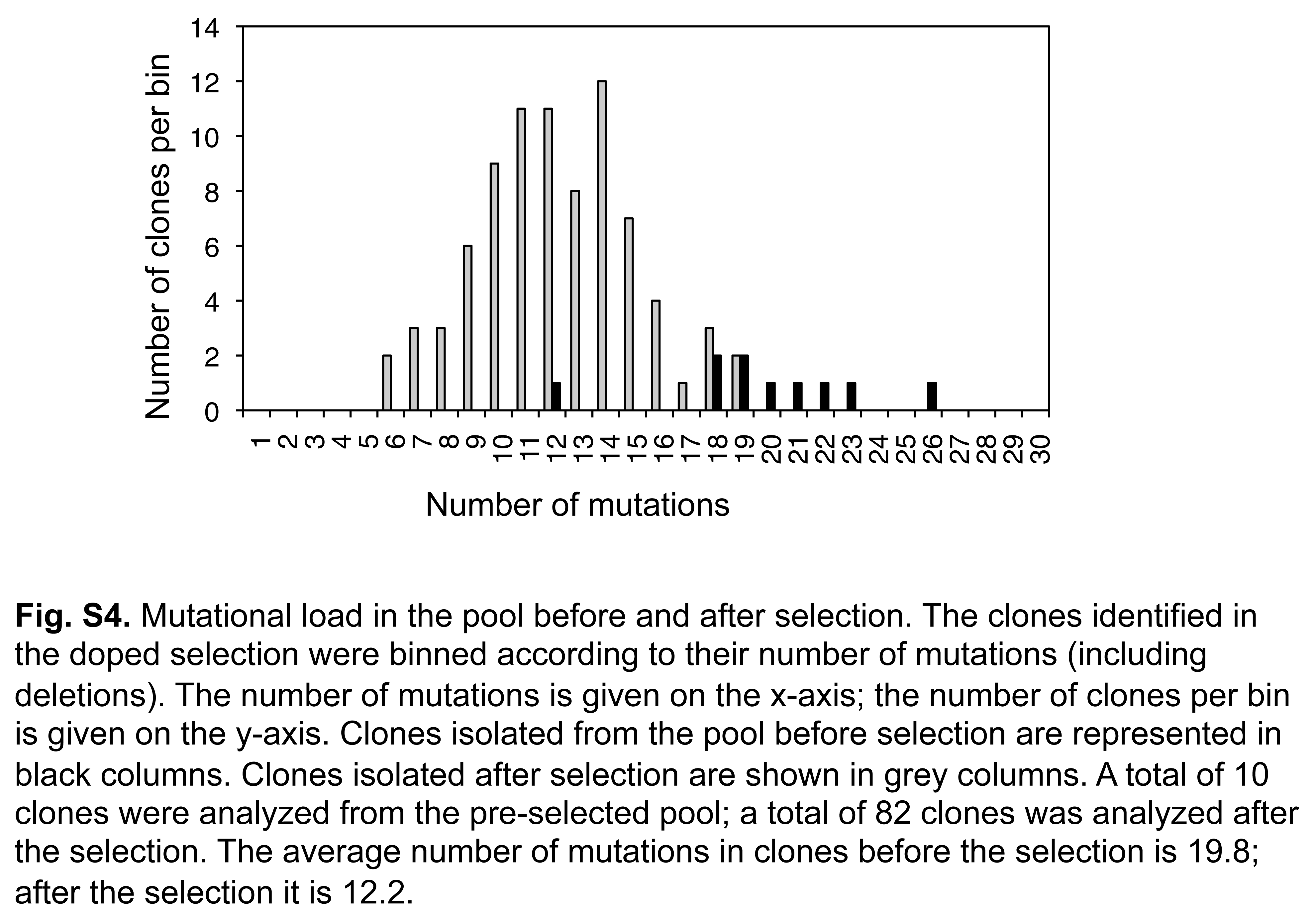

Supplement: S4 Fig — The clones identified in the doped selection were binned according to their number of mutations (including deletions). The number of mutations is given on the x-axis; the number of clones per bin is given on the y-axis. Clones isolated from the pool before selection are represented in black columns. Clones isolated after selection are shown in grey columns. A total of 10 clones were analyzed from the pre-selected pool; a total of 82 clones was analyzed after the selection. The average number of mutations in clones before the selection is 19.8; after the selection it is 12.2. (TIF) [file pone.0142559.s004.tif]
